# Analysis of head trauma management in a secondary hospital without neurosurgical service

**DOI:** 10.1186/cc10211

**Published:** 2011-06-22

**Authors:** M Steinman, L Lenci, C Kirschner, P Rogeri, S Possa, N Akamine

**Affiliations:** 1 Hospital Municipal Dr. Moyses Deutsch, Instituto de Ensino e Pesquisa, Hospital Israelita Albert Einstein, São Paulo - SP, Brazil

## Introduction

Head injuries are one of the most common causes of trauma patient admission. A key part of the management of these patients is airway control, rapid transport to appropriate trauma care facilities and prompt resuscitation. Many trauma patients who have suffered a head injury are initially taken to non-neurosurgical (NS) centers. In most instances, patients with severe head injury have to be transferred to a NS unit. Theoretically, the reason to transfer is the potential need for immediate surgical intervention. The purpose of the study was to evaluate head trauma patients who were transferred to NS units to determine the incidence of this occurrence, patients' profile and criteria adopted.

## Methods

A 6-month retrospective study was conducted from January through July 2010 at Hospital Municipal Dr. Moses Deutsch, located in Jardim Angela, south of São Paulo, 30 kilometers away from downtown. It is the only hospital within a radius of 7 miles and serves a population of approximately 600,000 inhabitants. It is a secondary hospital that provides medical staff in the emergency room for 24 hours as well as on-site computed tomographic (CT) scanning capability and the intensive care unit. All head trauma patients who were transported to NS were included. Data collected were demographics, mechanism of injury, Glasgow Coma Scale (GCS), clinical finding, CT findings, transfer times and returns from the NS.

## Results

There were 17,880 ED patient admissions and 2,255 were trauma related. A total 296 were head-injured patients requiring hospitalization. Eighteen seven patients demanded interhospital transfer, because of CT findings and clinical picture. The main mechanism of injury was falls (59.4%). The median transport delay to the neurosurgical service site was 10 ± 1.2 hours. Mean GCS were 12 and 56% of the CT had abnormal findings. Seventy-five percent returned after NS evaluation.

## Conclusion

Most of the cases are referred for assessment because of lack of local expertise leading to unnecessary transfers. This often resulted in the inappropriate transfer of ill patients and the unnecessary occupation of neurosurgical beds in a tertiary center. Furthermore, after assessment, many of these patients are sent back to the original hospital. Emergency neurosurgical teleconsultation may have an important role in the remote care of patients with head injuries and other neurosurgical emergencies.

